# Probiotics as multifaceted oral vaccines against colon cancer: A review

**DOI:** 10.3389/fimmu.2022.1002674

**Published:** 2022-10-03

**Authors:** Shubhi Singh, Manisha Singh, Smriti Gaur

**Affiliations:** ^1^ Department of Biotechnology, Jaypee Institute of Information Technology, Noida, India; ^2^ Department of Genomic Medicine, The University of Texas MD Anderson Cancer Center, Houston, TX, United States

**Keywords:** probiotics, mucosal immunity, colon cancer, oral vaccine, immunomodulators, antitumor, pathogenecity, PROBIO

## Abstract

Probiotics are known as the live microorganisms that, upon adequate administration, elicit a health beneficial response inside the host. The probiotics are known as immunomodulators and exhibit anti-tumor properties. Advanced research has explored the potential use of probiotics as the oral vaccines without the latent risks of pathogenicity. Probiotic-based oral vaccines are known to induce mucosal immunity that prevents the host from several enteric infections. Probiotic bacteria have the ability to produce metabolites in the form of anti-inflammatory cytokines, which play an important role in the prevention of carcinogenesis and in the activation of the phagocytes that eliminate the preliminary stage cancer cells. This review discusses the advantages and disadvantages of using the oral probiotic vaccines as well as the mechanism of action of probiotics in colon cancer therapy. This review also employs the use of “PROBIO” database for selecting certain probiotics with immunomodulatory properties. Furthermore, the use of several probiotic bacteria as anti-colon cancer adjuvants has also been discussed in detail. Because the current studies and trials are more focused on using the attenuated pathogens instead of using the probiotic-based vaccines, future studies must involve the advanced research in exploiting the potential of several probiotic strains as adjuvants in cancer therapies.

## Introduction

Cancer is more common today as a result of genetic abnormalities as well as epigenetic alterations in cell development and apoptotic pathways. Tumor development and growth are caused by the overexpression of genes associated with cell growth and proliferation in cancer and by the underexpression of genes that regulate cell death activities (1). Changes in cellular pathways are frequently produced by random mutations and unhealthy lifestyle habits. Immunocompetent individuals are vulnerable to natural immune alterations, which involve the activation of diverse immune responses to destroy competent tumor cells (2). Tumor-causing cells are going through apoptosis and necrosis as well as surrounding tissues are going through stress, and the microbiota in the gut all produce anti-cancer immune responses and signals.

Human gut microbes play pivotal role in regulating the immune responses, and the disturbance in gut microbiome often results in implication of several disorder including cancer (3). Since years, live vaccinations have been investigated as a therapeutic alternative to prevent cancer progression. Live vaccines have piqued researchers’ interest despite a number of safety issues, such as the reactivity to pathogenic organisms in immunosuppressed people. Research breakthroughs have contributed to the development of safer live vaccines and improved immunological approaches to combat the adverse effects of assimilation (4). In addition, focus is placed on using healthy gut microorganisms as the carriers for live vaccines. These beneficial gut bacteria are probiotics, which are described as live microorganisms that, when taken in sufficient quantities, benefit the host’s health. Probiotics have been known for altering the gut microbiota and triggering powerful immune responses in the host that are more effective at fighting cancer (5). The relationship between healthy gut microbes and colon cancer has been established as research on the gut microbiome develops.

One of the most prevalent malignancies is colon cancer, which is known to affect both men and women equally. Probiotics have been successfully used in recent years to alter the microbiome of the gut to treat colon cancer and reduce therapy-mediated side effects (6). Gut inflammation and neoplasia start to develop when the connection between the host’s immune system and probiotics is disrupted. Current studies and trials are more focused on development of vaccines using attenuated pathogens. These traditional vaccines have several disadvantages including the risk of conversion of attenuated forms back to virulent form inside the individual. Hence, there is a need to develop new form of safer vaccines. Probiotic-based vaccines offer several advantages over the traditional vaccines, which has been discussed in detail in the latter sections of this review.

This review paper discusses various benefits of probiotics as therapeutic agents. Along with this, mechanism of actions related to immunomodulatory effects exhibited by probiotics has also been comprehensively discussed. Probiotic bacteria and its fragments are responsible for generating several signals, activating various cytokines as well as apoptotic genes and eliciting an immune response. As it is known that probiotics release metabolites in the form of short-chain fatty acids (SCFAs) and extracellular proteins, these metabolites act as significant inhibitors of genes associated with tumor formation. Several attributes of probiotics as immunomodulators have also been comprehensively discussed in this paper. Anti-carcinogenic properties of probiotics are often indicated by the ability of probiotics to act as cytokine inducers, antigen presenters, and immuno-adjuvants. As there are several existing probiotic strains, there is a need to optimize the selection of only those strains exhibiting immunomodulatory properties. Hence, in this review, we have used the “PROBIO” database for selecting the probiotic strains with targeted properties. In addition to this, the opportunities as well as challenges involved in successful and effective consumption of probiotics as oral vaccine have also been reviewed.

## Benefits of probiotics as therapeutic agents in colon cancer

Probiotic bacteria are known to have beneficial effect on the overall immunity of the host. These bacteria are also responsible for eliciting anti-tumor responses with in the host. Major mechanisms involved in exhibiting the immunomodulatory benefits by probiotics have been discussed in this section. Along with this, “PROBIO” database has been employed for selecting and reviewing only those strains that are responsible for anti-tumor properties. Apart from this, various other strains that have already been used in minimizing the onset of colon cancer have been mentioned, and their anti-tumor effects have also been discussed in detail.

### Mechanisms for immunomodulatory effects by probiotics

Well-established links already exist between commensal bacteria and regulation of immune responses. Microbes, along with their bioactive metabolites, exert the immunomodulatory effects through one of the three proposed mechanisms (7):

- The metabolites secreted by probiotics help in modulation of systemic effects that include wound healing, fighting against primary infection and initiating recovery from distant tissues;
- regulation of immune responses by activation of pattern recognition receptors present on dendritic cells, macrophages, and antigen-presenting cells (APCs); and
- cross-reaction with tumor cell antigens and T cells, which helps in eliciting an immune response.

**Figure 1:**
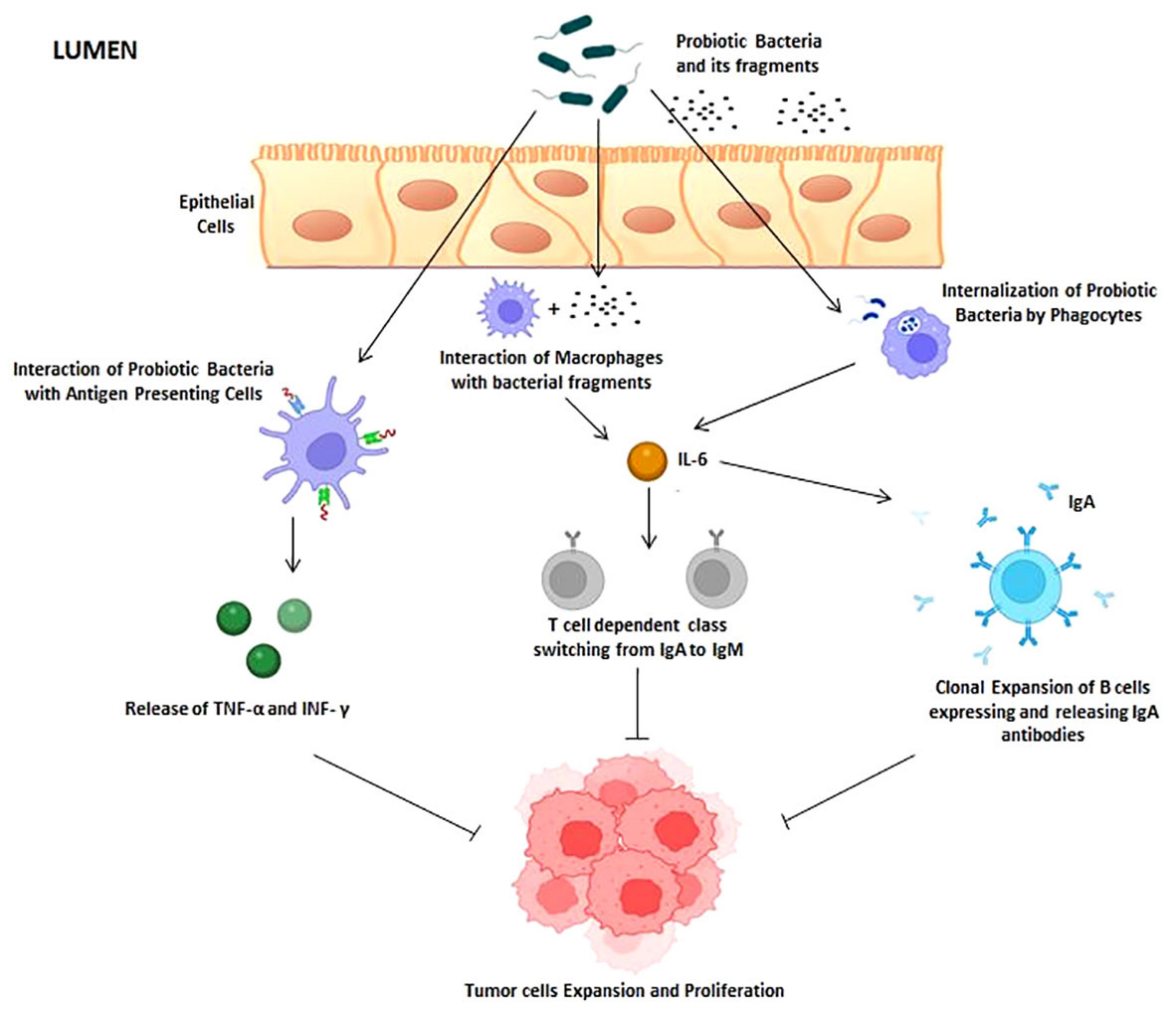
Interaction of probiotic cells with host immune system. Probiotic bacteria and its fragments are known to activate antigen-presenting cells (APC), macrophages (MQ), and phagocytes (PC). Once activated, these immune cells produce cytokines and interferon that result in inhibition of tumor expansion and proliferation.

In the human gut, bacterial cells interact with host cells such epithelial cells, macrophages, dendritic cells, as well as mucus-producing cells like goblet and paneth cells. These interactions lead to a cascade of signals that trigger an immune response. Interleukins (IL-2, IL-6, and IL-10) and the putative antimicrobial peptides defensins and cryptidins are also known to be secreted by mucus-producing cells (8, 9). Nitric oxide and other pro-inflammatory cytokines, such as tumor necrosis factor (TNF) and interferon (IFN), are also generated, enhancing the early innate immune responses by fostering the presentation of antigens. Early innate immune responses are activated by a combination of various signals. Epithelial cells, M cells, and dendritic cells assist in the internalization of the probiotic bacteria and its fragments into the lumen of the small intestine (10). After bacterial internalization, the bacterial cells interact with APCs, macrophages, and phagocytes, leading to release of cytokines like TNF-α and IFN-γ. Production of IL-6 is induced by the contact of non-pathogenic probiotic bacteria with epithelial cells. The number of IgA-expressing B cells is increased in the gut by IL-6, which also supports their clonal expression. In addition, IL-6, together with TGF, IL-4, and IL-10, and stimulates the T-dependent transition from immunoglobulin M (IgM) to immunoglobulin A (IgA) on B cells, resulting in an increase in the amount of IgA lymphocytes in the lamina propia of the gut (11) (**Figure 1**).

### Selection of probiotics with anti-tumor properties using potential database

Numerous probiotic bacteria, their metabolites, and other prebiotic components have been shown to influence colon cancer incidence and mediate gut immunity. Probiotics have the ability to modulate the immune system, but they also have other therapeutic benefits, such as the ability to reduce inflammation, prevent diarrhea, improve lactose tolerance, and exhibit anti-oxidative and anti-inflammatory properties (12). Furthermore, certain probiotic bacteria also have properties of clearing anti-nutritional metabolites as well as synthesis and enhanced absorption of vitamins and minerals (13). The probiotics originated from human gut have defined criteria of selection. Only those bacteria are selected, which helps in invasion of harmful pathogenic bacteria by establishing acidic environment and alleviates hypersensitivity reactions by modulating the host immunity (14). There are many different probiotic bacteria that can be used for therapeutic purposes; thus, it is important to explore how to best choose them through focused interventions. We used the “PROBIO” database (15), which includes information on 329 probiotics that are currently on the market, 115 probiotic bacteria that are undergoing clinical trials, and 118 probiotic bacteria that are currently being researched for their potential therapeutic benefits in humans. From the database, we have only selected particular probiotic bacteria with immunomodulatory capabilities as summarized in **Table 1**. It was observed that out of 20 potential probiotics, 12 probiotic strains have been successfully marketed for enhancement of immunity within the individuals. In addition to this, six probiotic strains are presently under clinical trial stage and two probiotic strains have been currently evaluated for their efficacy of immunomodulatory properties.

**Table 1:** Probiotic strains virtually screened from “PROBIO” database with potential immunomodulatory properties.

Probiotic bacteria	Strain	Status	Applications	References
*Bacillus amyloliquefaciens*	–	Research	Immune function in enhancement of phagocytosis by macrophages	(16)
*Bacillus polyfermenticus*	Bispan	Clinical trial	Enhancement of immune cells population IgG, CD4+ and CD8+ T cells, and NK cells	(17)
*Bifidobacterium animalis* subsp. *lactis*	BB-12	Clinical trial	Increases antigen-specific immune responses	(18)
*Bifidobacterium animalis* subsp*. lactis*	GPS1209	Marketed	Immune function enhancement	(15)
*Bifidobacterium animalis* subsp. *lactis*	HN019 (DR1064)	Clinical trial	Enhancement of innate immunity	(19)
*Bifidobacterium bifidum*	BB-12	Clinical trial	Increases antigen-specific immune responses	(20)
*Bifidobacterium bifidum*	Rosell-71	Marketed	Increases antigen-specific immune responses	(15)
*Bifidobacterium breve*	M-16V	Research	Enhances innate and adaptive immunity by increasing production of NF-κB	(21)
*Bifidobacterium longum*	–	Marketed	Enhancement of innate immunity	(15)
*Bifidobacterium thermophilum*	–	Marketed	Enhancement of innate immunity	(15)
*Lactobacillus acidophilus*	La-1	Clinical trial	Increases antigen-specific immune responses	(20)
*Lactobacillus brevis*	HA-112	Marketed	Enhances innate and adaptive immunity by increasing production of NF-κB	(15)
*Lactobacillus fermentum*	HA-179	Marketed	Increases antigen-specific immune responses	(15)
*Lactobacillus helveticus*	Lafti L10	Marketed	Increases antigen-specific immune responses	(15)
*Lactobacillus helveticus*	Rosell-52	Marketed	Enhancement of innate immunity	(15)
*Lactobacillus paracasei*	Lafti L26	Marketed	Enhancement of innate immunity	(15)
*Lactobacillus paracasei* subsp. *paracasei*	431	Clinical trial	Increases antigen-specific immune responses	(22)
*Lactobacillus rhamnosus*	HN001 (DR20)	Marketed	Enhancement of innate immunity	(15)
*Streptococcus salivarius*	DSM 13084	Marketed	Enhancement of innate immunity	(15)
*Streptococcus thermophilus*	–	Marketed	Enhancement of innate immunity	(15)

These applications are involved in enhancement of innate immunity and antigen-specific immune response and in increase of production of immune cells.

### Probiotics and colon cancer

Other than the probiotic bacteria mentioned in **Table 1**, various other probiotics have also been explored in animal tumor models to alleviate the onset of colon cancer represented in **Table 2**. Probiotics such as *Lactobacillus* and *Bifidobacteria* have been well researched for their medicinal benefits, among others. In one such study, the anti-cancer effectiveness of the putative probiotic bacterium *Akkermansia muciniphila* (AKK) was examined. For the investigation, tumor tissues from colon cancer patients were employed. In cancer-causing mouse models (B16F10 and CT26), the probiotic strain was combined with IL-2 therapy. The findings demonstrate that the combination of IL-2 and AKK improved tumor suppression in tumorigenic animal models. In addition, it was discovered that IL-2 activated Toll-like receptor 2 signaling receptors, which were responsible for the release of Amuc proteins from the outer membrane of probiotic bacteria (23). Probiotics and other bioactive substances are being investigated for their ability to prevent the development of cancer through chemically induced carcinogenesis in animal models. To give an example, the possible preventive effect against colon carcinogenesis was examined for *Lactobacillus rhamnosus* GG CGMCC 1.2134 (LGG). When a particular probiotic strain was administered to *in vitro* models that had previously been treated with 1,2-dimethylhydrazine dihydrochloride (DMH), it was shown that both the incidence and multiplicity of tumors were reduced. Likewise, probiotic supplementation to the tumorigenic models also resulted in reduction of inflammatory markers and anti-apoptotic proteins like nuclear factor kappa B (NF-κB–p65), cyclooxygenase-2 (COX-2), TNF-α, and B-cell lymphoma-2 (Bcl-2) and increased the expression of pro-apoptotic proteins like Bcl-2 Associated X protein (Bax), caspases-3 (casp3), and p53, when compared with control group (24). Yue and colleagues investigated further into anti-cancer benefits of oral supplementation of probiotic strain *Lactobacillus plantarum* strain YYC-3 and its cell-free supernatant form *Lactobacillus plantarum* strain YYCS in APCMin/+ mice models. The incidence of colon tumor and mucosal damage were found to be lowered by the intake of both probiotic strain types. In contrast to YCCS, which supported the suppression of the Wnt signaling pathway and the activation of NF-κB, YYC3 exhibited greater immunological responses by downregulating the expression of inflammatory cytokines IL-6, IL-17, and IL-22 (NF-κB) (25). Apart from using the single strains, the mixture of probiotics including *Lactobacillus acidophilus*, *Lactobacillus rhamnosus*, and *Bifidobacterium bifidum* was also used in evaluating the anti-cancerous properties. These strains had positive impact in attenuating the tumor growth and tumor size in azoxymethane/dextran sodium sulphate (AOM/DSS)–induced tumorigenic C57BL/6 mice models. Furthermore, the composition of the gut microbiota also changed as a result of an abundance of health-promoting bacteria living there, which decreased colitis and showed a nearly 50% decrease in the gut’s inflammatory index (26). Similar to this, in a different study, *Lactobacillus plantarum* increased the release of anti-inflammatory cytokines IL-10 and decreased the production of pro-inflammatory cytokines TNF-α and IL-8 in C57BL/6 mice treated with AOM/DSS. In addition, the administration of the chosen probiotic strain resulted in the elimination of harmful bacteria from the gut, including *Proteobacteria* and *Desulfovibrionaceae* (27). In lieu enhanced release of anti-inflammatory cytokines and attenuating the release of pro-inflammatory cytokines, these probiotics bacteria also indirectly support the prevention of colon cancer by increasing the health-benefiting microbial count in the gut. One such study by Wang and co-workers demonstrates that the supplementation of *Bifidobacterium bifidum* CGMCC 15068 in AOM/DSS-induced tumorigenic C57BL/6 mice models not only resulted in aattenuated incidence of tumorigenesis but also increased the microbial count of certain health-benefiting bacteria in the gut specifically *Verrucomicrobiaceae*, *Akkermansia*, and *Lactobacillus*. The gut microbiota manipulation with anti-cancerous properties is one promising preventive technique against colon cancers (28).

**Table 2:** Anti-tumor effects of probiotic strains in experimental *in vivo* models in managing colon cancer.

Probiotic bacteria	*In vivo* models	Experimental findings	References
*Akkermansia muciniphila*	B16F10 and CT26 tumor cells induced in mice models	• AKK + IL-2 supplementation enhanced the tumor suppression • Amuc protein from AKK activated Toll-like receptors signaling pathway	(23)
*Lactobacillus rhamnosus* GG CGMCC 1.2134 (LGG)	DMH-induced carcinogenesis in Sprague Dawley rats	• Reduced expression of anti-apoptotic proteins and inflammatory markers • Increased expression of pro-apoptotic proteins • Reduced tumor multiplicity and incidence	(24)
*Lactobacillus plantarum*	57BL/6-APC*Min/+* mouse models	• Reduced expression of pro-inflammatory cytokines • Downregulation of Wnt signaling pathway • Suppressed activation of NF-κB	(25)
*Lactobacillus acidophilus*, *Lactobacillus rhamnosus*, and *Bifidobacterium bifidum*	AOM/DSS-induced tumorigenic C57BL/6 mice models	• Reduced inflammatory index of gut • Reduced expression of TNF-α • Increased expression of IL-10	(26)
*Lactobacillus plantarum*	AOM/DSS-induced tumorigenic C57BL/6 mice models	• Reduced expression of pro-inflammatory cytokines • Increased expression of IL-10 • Elimination of pathogenic bacteria from gut	(27)
*Bifidobacterium bifidum* CGMCC 15068	AOM/DSS-induced tumorigenic C57BL/6 mice models	• Attenuated incidence of tumorigenesis • Increased the population of healthy bacteria in gut with anti-cancerous properties	(28)
*Bifidobacterium longum + Lactobacillus gasseri*	DMH-induced carcinogenesis in ICR mice	• Decreased the number of aberrant crypt foci • Reduced tumor multiplicity, tumor size and incidence • Increased macrophages which enhanced the release of PCNA and cyclin A	(29)
*Lactobacillus paracasei* DTA81	DMH-induced carcinogenesis in BALB/c mice	• Reduced expression of pro-inflammatory cytokines • Increased the release of short-chain fatty acid (SCFA)	(30)
*Lactobacillus fermentum* + *Lactobacillus plantarum*	DMH-induced carcinogenesis in Swiss Albino rats	• Decreased the number of aberrant crypt foci	(31)

These probiotic bacteria have been successfully evaluated for the anti-tumor effects against colon cancer. Experimental findings from the data strongly depict the increased production of anti-tumorigenic cytokines and the decreased production of pro-tumorigenic cytokines by probiotic bacteria.

## Attributes of probiotics in immunomodulation and anti-carcinogenesis

**Figure 2:**
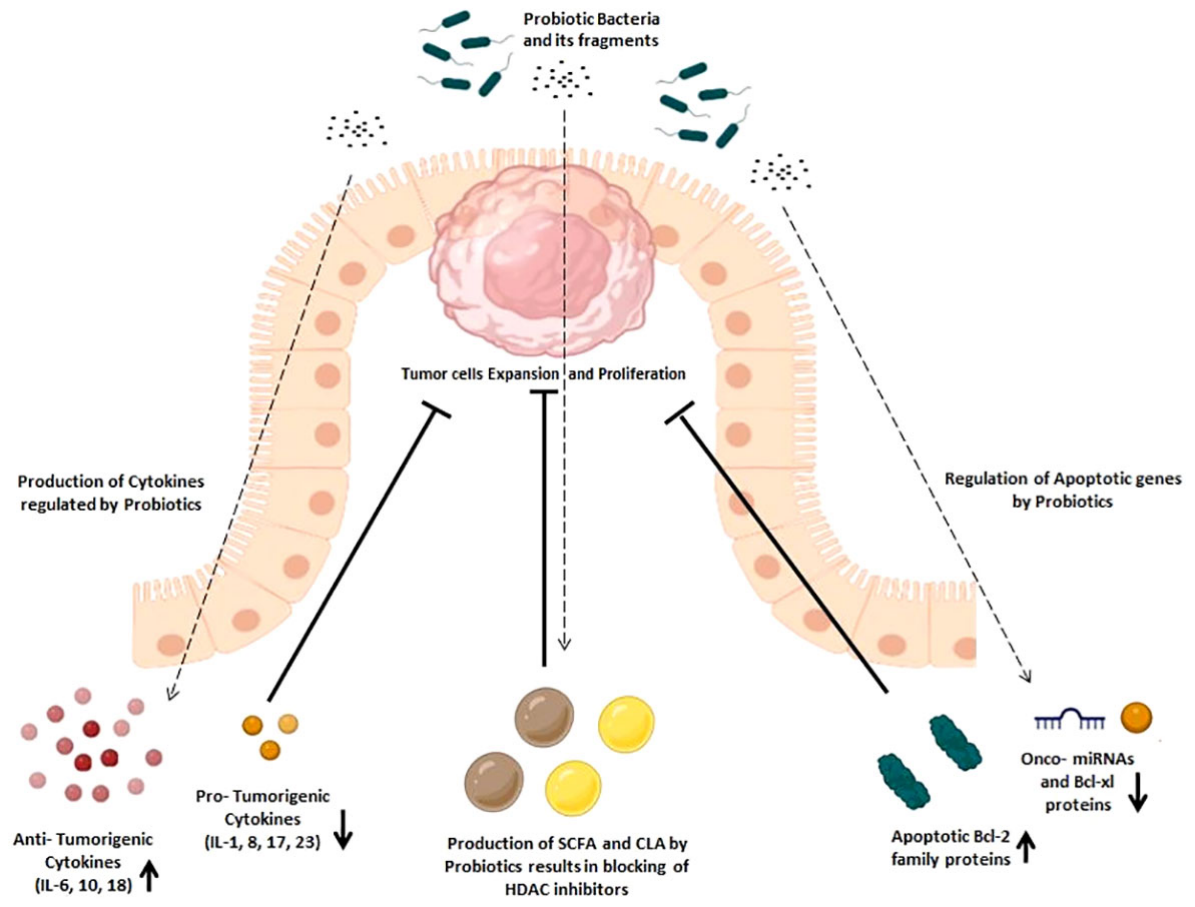
Immunomodulatory effects of probiotics in cancer prevention. Immunomodulatory effects are exhibited by the increased production of anti-tumorigenic cytokines and apoptotic Bcl-2 family proteins and the decreased production of pro-tumorigenic cytokines, onco-miRNAs, and Bcl-xl family proteins, which later helps in inhibition of tumor expansion and proliferation.

Escalating evidence indicates the possibility that a healthy microbiota may reduce the risk of colon cancer by delaying its onset. Probiotics and improved immune responses are closely associated, as shown by a number of cohort studies and ongoing clinical trials (32). By actively participating in or secreting specific metabolites that interact with Toll-like receptors to reinforce the epithelial intestinal barrier, probiotics are known to affect both cellular and immunological processes (33). In addition, probiotics also affect signal transduction pathways, leading to cytokine production and anti-inflammatory responses (34). In-depth discussion of probiotics’ molecular behavior in displaying their immunomodulatory and anti-carcinogenic characteristics is thoroughly covered this section (**Figure 2**).

### Cytokine production regulated by probiotics

Incidence of colon cancer is associated with impairment of immune system (35). Probiotics are known to induce the production of cytokines that enhance the immune system and help in alleviating the cancer cells progression. Moreover, probiotics are also known to stimulate the activity of phagocytic cells and the cytotoxic effects of natural killer cells (36). Cytokines, including ILs and IFN, are known to actively participate in the cell signaling pathways that help in elimination of cancerous cells (37). Probiotics have been explored for enhancing the production of anti-tumorigenic cytokine IL-18 that activates the CD8^+^ T cells (cytotoxic), Th1 macrophages, natural killer cells, and dendritic cells (38). One such study examined the anti-tumorigenic potential of a novel mixture of probiotic strains, including *Lactobacillus plantarum* VD23, *Lactobacillus plantarum* C28, *Lactobacillus plantarum* MS18, *Lactobacillus salivarius* MS3, *Lactobacillus salivarius* MS6, and *Lactobacillus salivarius* MS16, in chemically induced colon cancer in Wistar male rats. It was shown that adding certain probiotic strains to a diet slowed the growth of tumors and reduced their size and quantity. Furthermore, it was found that adding probiotics to the diet improved the production of IL-18 that stimulated the growth of epithelial cells, repaired mucosal cells, and enhanced release of antimicrobial peptides, all of which are significant in the prevention of carcinogenesis (39). Probiotic yeast has been used in the treatment of colon cancer as an alternative to *Lactobacillus* species. *Saccharomyces cerevisiae* was studied for its ability to prevent cancer in DSS-induced mice by Sun and colleagues. Probiotic bacteria supplementation was found to enhance the mucosal barrier and lessen the histological damage brought on by tumor growth. In addition, the expression of pro-inflammatory markers was diminished, while the histone acetylation and lactylation caused by the yeast metabolites contributed to the slowing of the development of cancer (40). On the other hand, *Lactobacillus casei* was given to BALB/c mice to examine its impact on cytokine production and anti-tumorigenic properties. Using Enzyme Linked Immuno Sorbent Assay (ELISA), the tumors’ cytokine production was examined, and it was found that the levels of cytokines such IL-10, IL-12p70, and IFN were elevated upon probiotics supplementation in the tumor cells that were collected from the spleen and Peyer’s patches. In addition, CD8^+^ cytotoxic T-cell production was enhanced accompanied by decline in tumor cell progression (41). Several probiotics have recently been studied in relation to Lipopolysaccharides (LPS)-induced inflammation in human colon cancer cell lines (HT29) and DSS-induced colitis in C57BL/6 mice. It was discovered that taking probiotic supplements not only reduced the synthesis of inflammation-related proteins and pro-inflammatory cytokines like IL-1 and IL-2 but also increased the production of anti-inflammatory cytokines like IL-10 and TGF- during the course of cancer (42).

### Metabolites produced by probiotics

Probiotic effectuates the fermentation of dietary fibers in gut and results in release of metabolites in the form of SCFA and conjugated linoleic acid (CLA) (43). SCFAs are produced as the end product of bacterial fermentation of partially and non-digested carbohydrates (44). Different fatty acids are produced depending upon the composition of intestinal microbiota, diet, and influential presence of other microbes in surrounding environment (45). These metabolites are consumed by colon cells as the energy source to further initiate apoptosis of cancerous cells, acidosis for removal of pathogenic bacteria, and regulation of other crucial enzymes. SCFAs have been reported to exert anti-carcinogenic properties in colon cancer progression (46). It is well recognized that butyrate is vital in preventing the progression of cancer and the cell cycle while also inducing the apoptosis of malignant cells (47). Furthermore, it has been noted that butyrate inhibits neuropilin-1, a gene regulator of apoptosis that is crucial for the expansion and migration of cancer cells (48). Vascular epithelial growth factor is known to bind to butyrate, which causes neuropilin to bind exclusively to the target receptor and modify its functional characteristics (49). By blocking the pro-apoptotic proteins Bax and Bak, which are members of the Bcl-2 family and are regarded as fundamental proteins involved in apoptotic pathways, butyrate influences the development of colon cancer in yet another way (50). Recent studies have examined into the potential involvement of butyrate, which is produced by the bacterium *Clostridium butyricum*, in reducing the risk of colon cancer. It was discovered that bacteria producing large levels of butyrate inhibited the growth and migration of tumor cells. The higher generation of butyrate also resulted in a considerable increase in the apoptotic activity of cancer cells, Wnt signaling pathways activity, and activation of G protein–coupled receptors (51). Another interesting study also reported the anti-proliferative behavior of butyrate on two colon cancer cell lines HCT116 and SW620. It was observed that butyrate reduced the cancer cell proliferation by downregulating the biomarkers. In addition to this, the degradation of catenin was observed in Wnt/β-catenin signaling pathways, which further resulted in reduction in transcriptional activity of cancerous cells (52).

Along with butyrate, CLA, a metabolite produced by probiotics, is regarded as being essential for slowing the progression of colon cancer (53). Probiotic bacteria in the intestine produce these compounds, which colon cells then absorb to have a positive local effect (54). Increased levels of specific probiotic bacteria in the gut are known to increase CLA synthesis, which inhibits G protein–coupled receptors and modulates signaling pathways (55). The ability of *Pediococcus pentosaceus* GS4 to produce CLA and its connection to the colon cancer cycle were examined by Dubey and his colleagues. In HCT-116 cell lines, it was found that the CLA produced by probiotic bacteria increased the apoptosis of malignant cells. In addition, the effects of CLA produced by these probiotic bacteria were investigated in mice with cancer caused on by AOM. According to the findings, CLA downregulated histone deacetylase (HDAC) activity, arbitrated signaling networks, and encouraged apoptosis in malignant colon cells (56). HDAC inhibitors play an important role in cancer progression through gene regulation by opening and closing the chromatin structures (57). The metabolites released by probiotics help in modulating the activity of HDACs by hyperacetylation of histones, which later inhibits the cancer cell progression and transportation by initiating apoptotic activities (58).

### Apoptotic genes regulation by probiotics

Probiotics have been explored in management of colon cancer by inducing its effects on regulatory genes and biomarkers involved in apoptosis (59). Probiotics’ critical role in controlling the cancer cell cycle has been studied in relation to miRNAs (60). In one such study, the anti-proliferative effects of *Lactobacillus acidophilus* were studied in Caco-2 cancer cell lines. It was observed that the involvement of probiotic strain resulted in inhibition of mRNA expression of apoptotic genes SURVIVIN and SMAC, hence proving the anti-proliferative properties of probiotic (61, 62). In one intriguing investigation, *Leuconostoc mesenteroides* with probiotic potential were isolated from dairy products. The beneficial effects of these microorganisms on regulating cancer cell apoptosis in HT-29 cell lines were studied. The findings demonstrated that probiotic bacteria can increase the activity of the Bax and Caspases, which, in turn, can promote apoptosis. B-cell lymphoma-extra-large (Bcl-xl), NF-kB, and other onco-micro RNAs (miRNA21 and miRNA 200b) were also all considerably downregulated (63). The ability of *Bifidobacterium longum* to regulate the activity of onco-micro RNAs has also been examined. Probiotic supplementation was found to increase the expression of miRNAs (miRNA 145 and miRNA 15a), which are crucial for tumor suppression, while decreasing the activity and production of onco-micro RNAs (miRNA 21a and miRNA 155) that are involved in cancer. The expression of anti-inflammatory cytokines was also controlled by these miRNAs, which also affected the growth of colon cancer in experimental animal models (64, 65). Chondrou and co-workers investigated the anti-proliferative effects of probiotic *Lactobacillus paracasei* K5 on Caco-2 cell lines. It was found that the isolated bacteria exhibited the increased expression of apoptotic Bcl-2 family proteins (66). It has been discovered that some probiotics, such as *Propionibacterium freudenreichii*, can successfully trigger apoptosis in HT-29 colon cancer cell lines through activation of TRAIL. TRAIL, also known as TNF-related apoptosis inducing ligand, is the substance that induces malignant cells to activate their extrinsic death mechanism. This investigation led the researchers to draw the conclusion that food-grade probiotic bacteria can serve as a supplement to help cancer cells begin to express apoptotic proteins (67, 68).

## Probiotics as oral vaccine: Opportunities, challenges and solutions

Traditional vaccination techniques and procedures target the systemic immunity of an individual (69). As probiotics exhibit several health benefits including the immunomodulation, the benefits of consuming these health beneficial bacteria as oral vaccines are a new area of research interest, which not only targets systemic immunity but also excels in managing the mucosal immunity (70). By employing probiotics as vaccine, the issue of systemic immunity failing to affect the inner mucosal layer of the colon is resolved (71). Probiotics penetrate the inner mucosal layer of the gut and aid in invasion of harmful bacteria, as well as protect against numerous enteric infections (72).

**Figure 3:**
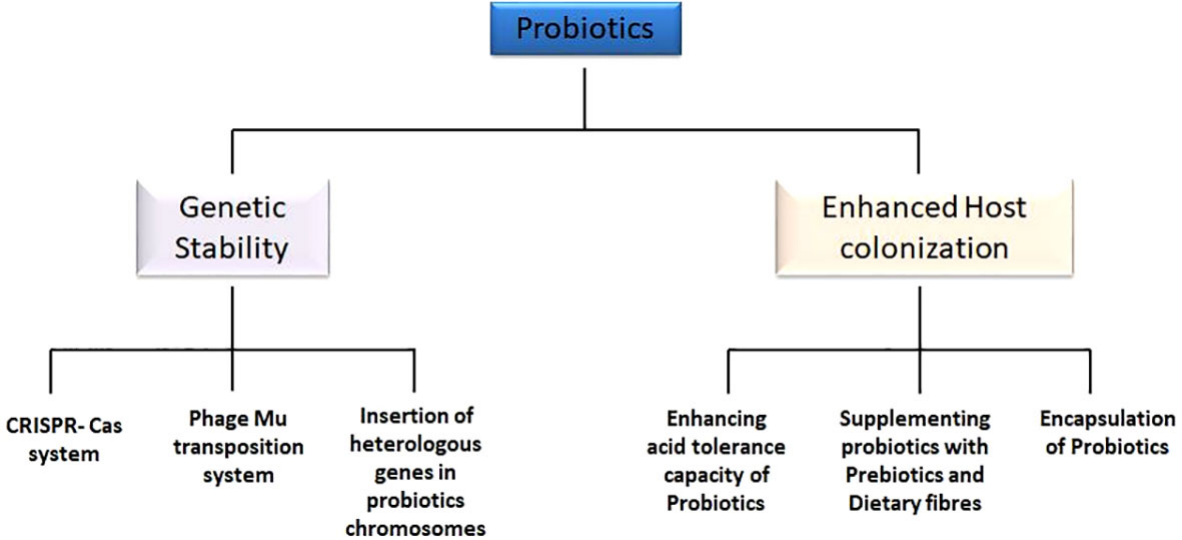
Challenges and current solutions to probiotic oral vaccines. Genetic stability and enhanced host colonization are major innovative strategies to overcome the challenges faced by probiotic vaccines. Genetic stability includes the insertion of heterologous genes in the bacterial chromosomes, adoption of CRISPR-Cas system, as well as Phage-Mu transposition system. Enhanced host colonization is achieved by enhancing acid tolerance capacity of probiotics, encapsulating probiotic cells, and supplementing probiotic cells with prebiotics and dietary fibers.

Moreover, unlike traditional vaccines, probiotic-based vaccines do not require critical storage conditions and transportation (73). Traditional live vaccines usually demand lower storage temperatures before administration (73, 74). In addition, traditional vaccinations are developed using attenuated strains of pathogenic bacteria, such as Salmonella, Mycobacterium, and Bacillus, which have a number of drawbacks, including the possibility of attenuated strains reverting to virulent forms inside the body (75). The attenuated forms of vaccines also result in eliciting additional immune response in individual, hence weakening the effect of vaccine. The survival rates under the harshly acidic conditions of the human gut are another issue that contributes to the failure of traditional vaccines as compared with probiotic oral vaccines (76). The traditional forms of vaccines are generally not able to survive the harsh acidic conditions, and, as a result, they fail to reach the inner mucosal layer. On the other hand, probiotics are well versed with the acidic environmental conditions. Because probiotics are acid- and bile-tolerant, they offer a good choice for developing vaccines (77). As probiotics offer several advantages over traditional vaccine, there exist some associated challenges in development of probiotics as oral vaccine. Some of the main issues with the development are the genetic instability and colonisation. However, recent research breakthroughs have led to several innovative solutions to the related problems (**Figure 3**).

### Genetic stability of probiotic-based oral vaccine

Probiotics can now be genetically modified due to advanced genetic technology; however, maintaining genetic stability is still perilous concern. Genetic alterations are described as the introduction of foreign genes that encode antigens or proteins into the recipient, followed by replication and expression (78). Several heterologous genes have been included into the bacterial chromosomes in an effort to reduce the genetic instability of probiotic vaccines (79). This helped in minimizing the plasmid replication burden on metabolic activities and also resulted in stable expression of inserted heterologous genes (80). For reduced genomic instability, phage-Mu–based transposition systems have also been frequently employed. This includes the formation of transposomes, which are defined as the complex of transposase protein MuA with DNA of the cell, which, on successful formation, results in cleavage and transformation of DNA (81). In addition to these approaches, CRISPR-Cas systems are the most effective and popular method for maintaining the genetic stability of probiotic vaccines. The CRISPRs are defined as short sequences (20–40 bp), which can be transcribed into crucial RNAs—transactivating crRNA and precursor crRNA (82). Together with guided RNA, these RNAs make up a crucial system that ensures stable insertion of the target gene into the host chromosome. To maintain genetic stability while developing oral vaccines, these techniques have been successfully applied in probiotic systems.

### Enhanced host colonization of probiotic-based oral vaccine

It is crucial to develop defensive system mechanisms for probiotic vaccines to survive harsh gut conditions because probiotics must persist in the human gut for a longer period of time to manifest their beneficial properties. Several *Bifidobacterium* and *Lactobacillus* species have demonstrated acid tolerance properties (83, 84). Thus, modifying specific probiotics to make them acid-tolerant is one way to achieve enhanced host colonization of probiotic vaccines. Making probiotics more acid-tolerant has been accomplished through a number of mechanisms and methods, such as biofilm formation, malolactic fermentation, the arginine dihydrolase system, DNA repair and protection, and amino acid decarboxylation (79). In addition to these methods, some researchers have also introduced the acid-tolerant gene in probiotics using genetic engineering methods for improved survival (85). The probiotic oral vaccines have also been supplemented with additional prebiotics compounds such as non-digestible dietary fibers that act as the energy source and aids probiotics in longer survival under harsh conditions (86). Probiotic bacteria vaccines have also been protected using a variety of encapsulation techniques. Recent research focuses on the expression of adhesion proteins in *Lactobacillus* and *Bifidobacteria* to improve host colonization (87). For instance, other probiotic strains have successfully expressed Bifidobacterium adhesion proteins like BopA, which improved their ability to colonize the gut’s epithelial layer (88).

## Conclusion and future prospects

Cancer is still a serious concern and a major reason for mortality around the world. High-cost chemotherapies, surgeries and traditional forms of vaccination are also an issue of economic concern and increase the chances of several side effects. Hence, more attention is now focused on probiotic-based oral vaccines. From several studies evidenced in this review, commensal healthy bacteria and probiotics exert influential effects by immunomodulatory and metabolic activities on host. A slight disturbance in gut microbiome and its functions may result in onset of colon carcinogenesis. These may be regarded as beneficial in management of cancer either in early stages of tumor formation or during the period of cancer therapy. Consequently, the use of probiotic rich food may help in diminishing the risk of developing colon cancer. Today, a great interest encourages scientific researchers to explore the potential of several probiotic strains to induce apoptosis as a promising approach for managing colon carcinogenesis. Through the research studies mentioned in this paper, it is known that several probiotic bacteria manage to control the carcinogenesis by cytokine production, metabolites production, and regulation of apoptotic genes and to reduce the inflammation in gut during cancer. However, the effect of individual bacteria upon cancer prevention is distinctive. Oral vaccination with active or attenuated probiotic strains has several advantages with non-invasive and non-pathogenic characteristics, as compared with traditional vaccines.

However, following the mentioned *in vivo* and *in vitro* studies, further research explorations must be considered as clinical trials to fully determine the anti-cancer properties of probiotic-based vaccines. Otherwise, extensive human trials are still needed to confirm the efficacy of this approach. Indeed, clear experiments must be designed to evaluate the beneficial and adverse effects of probiotics vaccines. Future studies must also include more critical research in understanding the molecular mechanisms of other novel probiotic strains isolated from functional dairy and non-dairy food products, hence narrowing the gap between food sector and pharmaceutical sector in the coming future.

## Author contributions

SS and SG contributed to conception and design of the study. SS, MS and SG wrote the first draft of the manuscript. All authors contributed to manuscript revision and read and approved the submitted version.

## Funding

This work was supported by the University Cancer Foundation via the Institutional Research Grant program at the University of Texas MD Anderson Cancer Center (to MS).

## Conflict of interest

The authors declare that the research was conducted in the absence of any commercial or financial relationships that could be construed as a potential conflict of interest.

## Publisher’s note

All claims expressed in this article are solely those of the authors and do not necessarily represent those of their affiliated organizations, or those of the publisher, the editors and the reviewers. Any product that may be evaluated in this article, or claim that may be made by its manufacturer, is not guaranteed or endorsed by the publisher.
